# Impact of surgeon work duration prior to distal pancreatectomy on perioperative outcomes: a propensity score matching analysis

**DOI:** 10.1186/s12893-021-01062-0

**Published:** 2021-01-22

**Authors:** Zhen Wan, Xuzhen Wang, Yong Li, Renhua Wan

**Affiliations:** 1 Department of General Surgery, The First Affiliated Hospital of Nanchang University, Nanchang, 33006 China; 2 Department of Critical Care Medicine, The First Affiliated Hospital of Nanchang University, Nanchang, 330006 China

**Keywords:** Work duration, Fatigue, Distal pancreatectomy, Propensity score matching

## Abstract

**Background:**

Surgeons are likely to get progressively fatigued during the course of a normal workday. The objective of this study was to evaluate the impact of surgeon work duration prior to performing distal pancreatectomy (DP) on the perioperative outcome, especially frequency of grade II or higher grade postoperative complications.

**Methods:**

Patients undergoing DP for all causes were divided into two groups according to surgeon work hours prior to performing DP: group A (less than 5 h) and group B (5–10 h). Propensity score matching (PSM) analysis (1:1) were performed to balance the baseline characteristics between the two groups. Intraoperative complications were compared between the two groups. Postoperative complications and their severity were followed up for 60 days and mortality for 90 days. The study was powdered to identify a 15% difference in the incidence of grade II or higher grade complications.

**Results:**

By using PSM analysis, the patients in group A (N = 202) and group B (N = 202) were well matched regarding demographics, comorbidities, operative technique, pancreatic texture and pathology. There was no significant difference in the incidence of grade II or higher grade complications between the two groups. There was no difference in clinically relevant postoperative pancreatic fistula, percutaneous drainage, readmission, reoperation, or morality. Group B was associated with a higher incidence of intraoperative organ injury, which could be managed successfully during the operation.

**Conclusion:**

The retrospective study demonstrated that the surgeon work duration did not significantly affect the clinical outcome of DP.

## Introduction

Surgeons are likely to experience physical and mental fatigue during the course of a normal workday [1]. Surgeon fatigue due to long working hours may result in decreased surgical performance and worse patient outcome [2]. It was reported that afternoon colonoscopies had a significantly lower adenoma detection rate than morning colonoscopies [3, 4]. General and vascular surgical operations starting between 4 and 6 p.m. were associated with an elevated risk of morbidity over those starting between 7 am and 4 p.m. [5]. Previous research also demonstrated that surgeon fatigue was an important factor contributing to intraoperative errors [6]. However, some other studies have not replicated these findings. Surgery start time during the work day had no measurable influence on patient outcome following pancreaticoduodenectomy [7], liver resection [8] and laparoscopic colectomy [9].

Distal pancreatectomy (DP) is a complex, technically demanding procedure with high complication rates [10]. Due to different complication profiles, DP and other surgeries deserve separate evaluations. To the best of our knowledge, the impact of work duration on the perioperative outcome after DP has not been reported before. The aim of the study was to examine the potential effects of surgeon work duration prior to performing DP on the frequency of grade II or higher grade postoperative complications.

## Method

### Patients and data collection

Patients receiving DP in First Affiliated Hospital of Nanchang University from January 2010 to July 2020 were included. All DP procedures included in this study were performed by two board-certified pancreatic surgeons (Li Y and Wan RH) with an experience of 500 cases of pancreatic surgery within the previous 10 years. Patients undergoing multivisceral resection, emergency or robotic surgery were excluded from the current study. All eligible patients were divided into group A (less than 5 h) or group B (5–10 h) in terms of surgeon work duration prior to performing DP. Before performing DP, surgeons usually treated patients in outpatient department, performed other operations or taught the trainees during the work time. This study was undertaken in accordance with the principles of the Declaration of Helsinki and was approved by the ethics committee of the First Affiliated hospital of Nanchang University. A waiver of informed consent was obtained, since the data were analyzed from the electronic medical record and reported without personal identifiers.

Patient characteristics consisted of gender, age, body mass index (BMI), comorbidities (cardiac and pulmonary disease, diabetes mellitus, hypertension and renal insufficiency), tobacco use and American Society of Anesthesiologists (ASA) physical status score. Laboratory data such as blood routine examination, liver and renal function tests were recorded prior to DP. Surgical approach (laparoscopic or open), procedure performed (standard or spleen-preserving DP) and pancreatic texture estimated by surgeon (soft or hard) were collected from the operation records. Pathological diagnosis (benign or malignant) was obtained from the analysis of resection specimens.

Operation time, estimated intraoperative blood loss and red blood cell transfusion requirement data were extracted from the anesthesia records. Operation time was defined as the time from incision to application of the final wound dressing. Intraoperative complications included unnecessary damage to adjacent vessels and organs during DP. Intraoperative complication was graded using the Satava approach [11]: Grade I, incidents managed without change of operative approach and without further consequences for the patient. Grade II, incidents with further consequences for the patient, including resection of injured organs and intraoperative blood loss over normal range. Grade III, incidents leading to significant consequences for patient. Length of hospital stay (LOS) was measured as the number of days from operation to discharge. Postoperative percutaneous drainage and reoperation were recorded in the hospital daily progress notes. Readmission was defined as an admission to the hospital for 24 h or more within 60 days after surgery. Mortality occurring within 90 days of surgery was documented.

### Definition and classification of postoperative complications

Clinically relevant postoperative pancreatic fistula (Grade B/C) referred to those requiring prolonged drainage, reoperation, and/or death, while Grade A was no longer considered as a true postoperative pancreatic fistula (POPF) according to the criteria of the International Study Group [12]. Postpancreatectomy hemorrhage (PPH) and delayed gastric emptying (DGE) were also identified using the schema proposed by the International Study Group of Pancreatic Surgery [13, 14]. Abdominal infection was diagnosed by the presence of signs of peritonitis, leukocytosis, and/or positive drainage fluid culture. All complications occurring within 60 days of surgery were recorded and classified into five grades (grade I-V) using the Clavien-Dindo classification system [15]. The primary outcome measure for this study was grade II or higher grade complication rate.

### Propensity score matching (PSM)

PSM method was applied to balance the baseline characteristics of the two groups. Gender, age, BMI, comorbidities, tobacco use, ASA score, albumin level, hemoglobin level, serum creatinine level, surgical approach, surgical procedure, pancreatic texture and pathology were used as covariates in the propensity score analysis. 1:1 matching without replacement was performed using a caliper of 0.02, and the resulting score-matched pairs of patients were used in subsequent analyses.

### Statistical analysis

All statistical analyses were performed using SPSS 22.0 software (Chicago, IL). Data were expressed as mean $\pm$ standard deviation, number (percentage), or median (interquartile range). Categorical variables were compared using the chi-square test or Fisher exact test as appropriate. For numerical variables, the *t* test or Wilcoxon rank sum test was used. 2-sided statistical tests were used and a *P* value less than 0.05 was considered statistically significant.

The appropriate sample size was calculated based on the assumption of a difference of 15% in grade II or higher grade complication rate between the two groups [16]. 342 evaluable patients were needed to detect this difference (α set at 0.05; β set at 0.2; power = 80%).

## Results

**Fig. 1:**
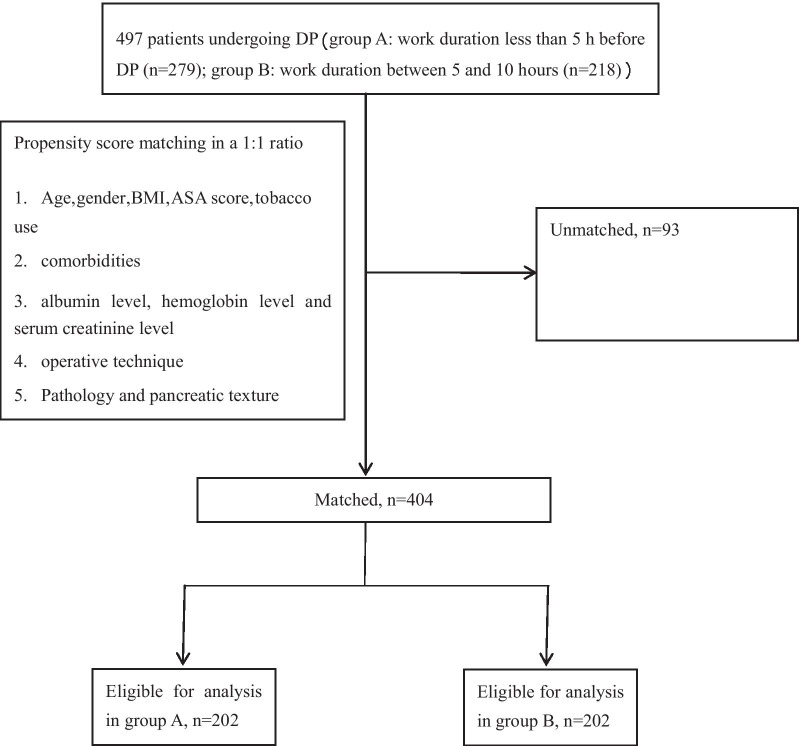
Flowchart of patient selection

A total of 497 patients undergoing DP were enrolled in this study (Fig. 1). Patients were allocated into two groups according to the work duration of surgeons prior to DP: Group A (n = 279) and Group B (n = 218). The baseline characteristics for the two groups of patients were shown in Table 1. Patients in group A had a lower BMI than those in group B (*p* < 0.001). ASA physical status score seemed to be higher in group A than group B (*p* = 0.015). After propensity score matching, there was no significant difference in any patient characteristics between Group A (n = 202) and Group B (n = 202). (*p* > 0.05, Table 2).

**Table 1:** Baseline characteristics of 497 patients undergoing DP

Variables	Group A (n = 279)	Group B (n = 218)	*p* value
Gender (male)	118 (42.3)	105 (48.2)	0.192
Age (years)	60.4 $\pm$ 6.6	61.0 $\pm$ 5.9	0.281
Body mass index (kg/m^2^)	28.9 $\pm$ 2.4	29.7 $\pm$ 2.5	< 0.000
Comorbidities			
Hypertension	126 (45.2)	90 (41.3)	0.387
Diabetes mellitus	55 (19.7)	41 (18.8)	0.800
Cardiac and pulmonary diseases	19 (6.8)	12 (5.5)	0.550
Renal insufficiency	15 (5.4)	11 (5.0)	0.870
Tobacco use			0.503
Current	45 (16.1)	44 (20.2)	
Former	103 (36.9)	76 (34.9)	
Never	131 (47.0)	98 (45.0)	
ASA			0.015
1 + 2	123 (44.1)	120 (55.0)	
3 + 4	156 (55.9)	98 (45.0)	
Albumin (mg/dL)	40.6 $\pm$ 2.7	40.2 $\pm$ 2.8	0.053
Hemoglobin (g/dL)	13.6 $\pm$ 0.8	13.5 $\pm$ 0.7	0.639
Creatinine (mg/dL)	0.8 $\pm$ 0.1	0.8 $\pm$ 0.1	0.078
Surgical approach			0.590
Open DP	173 (62.0)	130 (59.6)	
Laparoscopic DP	106 (38.0)	88 (40.4)	
Splenectomy			0.739
Standard DP	236 (84.6)	182 (83.5)	
Spleen preserving DP	43 (15.4)	36 (16.5)	
Pancreatic texture			0.586
Soft	196 (70.3)	158 (72.5)	
Hard	83 (29.7)	60 (27.5)	
Pathology			0.426
Benign	150 (53.8)	125 (57.3)	
Malignant	129 (46.2)	93 (42.7)

**Table 2:** Comparison of baseline characteristics of patients after PSM

Variables	Group A (n = 202)	Group B (n = 202)	*p* value
Gender (male)	94 (46.5)	93 (46.0)	0.921
Age (years)	61.0 $\pm$ 6.4	61.1 $\pm$ 6.0	0.853
Body mass index (kg/m^2^)	29.5 $\pm$ 2.2	29.4 $\pm$ 2.3	0.495
Comorbidities			
Hypertension	88 (43.6)	85 (42.1)	0.763
Diabetes mellitus	34 (16.8)	38 (18.8)	0.603
Cardiac and pulmonary diseases	13 (6.4)	12 (5.9)	0.836
Renal insufficiency	11 (5.4)	9 (4.5)	0.646
Tobacco use			0.940
Current	35 (17.3)	37 (18.3)	
Former	72 (35.6)	69 (34.2)	
Never	95 (47.0)	96 (47.5)	
ASA			0.842
1 + 2	103 (51.0)	105 (52.0)	
3 + 4	99 (49.0)	97 (48.0)	
Albumin (mg/dL)	40.4 $\pm$ 2.7	40.1 $\pm$ 2.8	0.332
Hemoglobin (g/dL)	13.5 $\pm$ 0.8	13.5 $\pm$ 0.7	0.916
Creatinine (mg/dL)	0.8 $\pm$ 0.1	0.8 $\pm$ 0.1	0.809
Surgical approach			1.000
Open DP	124 (61.4)	124 (61.4)	
Laparoscopic DP	78 (38.6)	78 (38.6)	
Splenectomy			0.590
Standard DP	167 (82.7)	171 (84.7)	
Spleen preserving DP	35 (17.3)	31 (15.3)	
Pancreatic texture			0.717
Soft	157 (77.7)	160 (79.2)	
Hard	45 (22.3)	42 (20.8)	
Pathology			1.000
Benign	113 (55.9)	113 (55.9)	
Malignant	89 (44.1)	89 (44.1)	

Surgical outcomes including operating time, LOS, instances of postoperative percutaneous drainage, readmission, reoperation and 90-day mortality were comparable between the two groups (*p* > 0.05, Table 3). One patient in group A died of progressive multiple organ failure due to pancreatic fistula, while two patients in group B died of abdominal hemorrhage and hemorrhagic shock secondary to fistula. The estimated intraoperative blood loss in group A was higher than that in group B (*p* < 0.000). However, rate of red blood cell transfusion did not differ significantly between the two groups. The incidence of intraoperative organ injury was lesser in Group A than in Group B (*p* = 0.023), while intraoperative vascular injury did not significantly differ between the two groups.

**Table 3:** Surgical outcomes

Variables	Group A (n = 202)	Group B (n = 202)	*P* value
Operating time (min)	175 (160.0–190.0)	172 (158.0–182.3)	0.148^#^
Estimated blood loss (ml)	232.2 $\pm$ 91.0	198.3 $\pm$ 91.9	< 0.000
Red blood cell transfusion	21 (10.4)	18 (9.0)	0.613
Intraoperative complications	18 (8.9)	29 (14.4)	0.088
Adjacent organ injury	9 (4.5)	21 (10.4)	0.023
Vascular injury	9 (4.5)	9 (4.5)	1.000
Vascular injury (Grade II)	3 (1.5)	2 (1.0)	1.000*
Length of hospital stay (days)	8.6 $\pm$ 2.1	8.8 $\pm$ 2.8	0.488
Postoperative percutaneous drain	22 (10.9)	19 (9.4)	0.742
Hospital readmission	9 (4.5)	7 (3.5)	0.800
Reoperation	3 (1.5)	5 (2.5)	0.724*
90-day morality	1 (0.5)	2 (1.0)	1.000*

*Fisher exact test, ^#^Wilcoxon rank sum test.

116 patients in group A and 117 patients in group B experienced one or more perioperative complications respectively (*p* = 1.000). There was no difference between the two groups with respect to the study’s primary outcome variable: the number of patients with grade II or higher grade complication. The percentage of these patients was 40.6% in group A and 45.5% in group B (*p* = 0.366, Table 4). There was also no difference in the number of patients with grade III or higher grade complication and quantity of complications per patient between the two groups.

**Table 4:** Prevalence and severity of postoperative complications 60 days after DP

Variables	Group A (n = 202)	Group B (n = 202)	*p* value
Any complication	116 (57.4)	117 (57.9)	1.000
Any>>grade II complication	82 (40.6)	92 (45.5)	0.366
Any>>grade III complication	45 (22.3)	41 (20.3)	0.716
Median number of complication	1 (0–2)	1 (0–2)	0.520^#^

^#^Wilcoxon rank sum test

We further compared the incidence of postoperative complications between the two groups. There was no significant difference in clinical relevant PORF and abdominal infection between the two groups (Table 5). The incidence of PPH and DGE was equal between the two groups as well (*p* = 0.823, 0.739, respectively). There was also no difference in any other complications such as wound infection, thromboembolic event, cardiac complication, pulmonary complication, urinary tract infection and urinary retention.

**Table 5:** Postoperative complications 60 days after DP

Variables	Group A (n = 202)	Group B (n = 202)	*p* value
Clinically relevant pancreatic fistula	31 (15.3)	39 (19.3)	0.293
Postpancreatectomy hemorrhage	10 (5.0)	11 (5.4)	0.823
Delayed gastric emptying	21 (10.4)	19 (9.4)	0.739
Abdominal infection	22 (10.9)	28 (13.9)	0.365
Wound infection	11 (5.4)	10 (5.0)	0.823
Thromboembolic event	2 (1.0)	1 (0.5)	1.000^*^
Pulmonary complication	15 (7.4)	17 (8.4)	0.713
Cardiac complication	20 (9.9)	23 (11.4)	0.628
Renal insufficiency	6 (3.0)	7 (3.5)	0.778
Urinary retention	12 (6.0)	13 (6.4)	0.836
Urinary tract infection	7 (3.5)	9 (4.5)	0.610

*Fisher exact test

## Discussion

The retrospective study evaluated the influence of surgeon work duration on the perioperative outcome after DP using PSM analysis. Clavien-Dindo Grade II or higher grade complication rate was comparable between the less than 5 h group (group A) and the 5–10 h group (group B). Further analysis demonstrated no significant difference in the incidence of main postoperative complications including clinical relevant PORF, PPH, DGE, and abdominal infection.

The study indicated that the incidence of intraoperative organ injury was significantly higher in group B than that in group A. However, there was no difference in operating time and LOS between the two groups. Procedure related complications such as gastric perforation, colocolic fistula and abdominal hemorrhage were also not observed postoperatively. The results suggested the adjacent organ injury could be managed successfully during the operation. Interestingly, intraoperative vascular injury was comparable between the two groups.

The relationship between surgeon work duration and short-term outcome of patients has not been fully elucidated. It seems plausible that long work hours may be associated with poor clinical outcome and increased morbidity and mortality rates. Even in relatively simple procedures such as colonoscopies [4] and cardioverter-defibrillator implantation [17], worse results were observed in the afternoon group than in the morning group. It may be attributed to physicians fatigue after several hours’ work. However, other studies revealed that whether the surgeons had performed other surgeries prior to complex liver resection [8] or prostatectomy [18] in the same day did not affect the perioperative outcome.

In this study, the incidence and severity of postoperative complications after DP in group B was not significantly higher than that in group A, suggesting that long working hours of surgeons did not lead to a poor patient outcome. There are several possible explanations for the null results. First, DP combined with multivisceral resection was not included in this study due to enhanced invasiveness and higher risks of complications compared to standard DP or spleen preserving DP. The median operating time for DP combined with multivisceral resection reported exceeds 5 h, and surgeons are prone to lose concentration and work less effectively [19]. Second, DP was performed by experienced attending surgeons rather than residents in our center, which may reduced the adverse effects of fatigue on surgeon performance greatly. Senior resident surgeon could accomplish many procedures such as laparotomy, exploration and transaction of gastrocolic ligament excellently under the supervision of experienced surgeons [20], while the attending surgeon may have a short rest to alleviate fatigue. Third, procedures of distal pancreatectomy were standardized in our center to reduce the incidence of surgical errors. Pancreatic fistula is believed to contribute to the most morbid complications including retroperitoneal vascular erosion, abdominal hemorrhage, intra-abdominal abscess, sepsis, multiple organ failure, and even death. Pancreatic fistula is closely related to surgical resection method and closure technique of the pancreatic remnant [21]. Stapler resection followed by laparoscopic reinforcement suture of pancreatic stump was applied in our center with a low incidence of pancreatic fistula [22]. A retropancreatic tunnel was created and the pancreatic parenchyma was transected using a linear stapler. Then the pancreatic stump was sutured by 4/0 polypropylene and a suction drain was positioned in the splenic fossa close to the transected pancreas.

The effect of surgeon work duration on mortality after DP should be analyzed with caution. This study revealed that there was no significant difference in mortality rate between the two groups. However, this study did not have adequate statistical power to detect the difference in mortality rate as death after DP occured rarely. One patient in group A (0.5%) and two patients in group B (1%) died during 90 days after the surgery, respectively. Thus, we could not conclude certainly that long work duration of surgeon increases the mortality rate after DP.

The major limitation of the present study is that it is a retrospective study, which might cause sample selection bias. PSM analysis was performed to match demographics, comorbidities, pathology and pancreatic texture of the two groups. There was also no significant difference in surgical approach (laparoscopic versus open) and procedure performed (standard versus spleen-preserving DP), which eliminated the influence of surgery-related factors on perioperative parameters and patient outcome. Robotic DP was excluded from this study as robotic DP was mainly scheduled in the night and those patients could not be matched. However, the 402 patients enrolled in the retrospective study were performed in the past 10 years and the long period of accrual may affect the outcome of the study.

## Conclusion

The objective of this study was to evaluate the effect of surgeon work duration before DP on the perioperative outcome. The retrospective study revealed that surgeon work duration prior to performing DP did not affect the incidence of grade II or higher grade complications. Although incidence of intraoperative organ injury was higher after longer work duration, adjacent organ injury could be managed successfully during the operation.

## Data Availability

The datasets used and/or analysed during the current study are available from the corresponding author on reasonable request.
